# Improved functional outcomes and cost benefits of door-to-needle time under 30 min in acute ischemic stroke: an observational study

**DOI:** 10.3389/fstro.2025.1583875

**Published:** 2025-06-03

**Authors:** Jia Dong James Wang, Ying-Qiu Dong, Joshua Y. P. Yeo, Kevin Soon Hwee Teo, Shiyang Ng, Mingxue Jing, Bernard P. L. Chan, Leonard L. L. Yeo, Magdalene L. J. Chia, Louis Widjaja, Lily Y. H. Wong, Pamela Lim, Shikha Kumari, Diarmuid Murphy, Hock-Luen Teoh, Benjamin Y. Q. Tan

**Affiliations:** ^1^Lee Kong Chian School of Medicine, Nanyang Technological University, Singapore, Singapore; ^2^Value Driven Outcomes Office, Academic Informatics Office, National University Health System, Singapore, Singapore; ^3^Division of Neurology, Department of Medicine, National University Hospital, Singapore, Singapore; ^4^Department of Medical Affairs, National University Hospital, Singapore, Singapore

**Keywords:** stroke, ischemic stroke, thrombolysis, functional status, outcome assessment, healthcare costs, patient outcome assessment, outcome and process assessment

## Abstract

**Introduction:**

Intravenous thrombolysis (IVT) is cornerstone of acute ischemic stroke(AIS) recanalization therapy. Clinical guidelines advocate achieving Door-to-Needle (DTN) time of 60 min or less, with recent evidence highlighting clinical advantages of even shorter DTN times. However, economic implications of reducing DTN time are less well-studied. This study aims to assess shorter DTN targets impact on clinical outcomes and healthcare costs.

**Methods:**

This observational cohort study included consecutive patients with AIS treated with IVT in a comprehensive stroke center from January 2017 to December 2023. Patients were stratified by DTN time into 4 groups: ≤ 30, 31–45, 46–60, and >60 min. Multivariate linear and logistic regressions were performed to evaluate impact of DTN time on functional and financial outcomes, including modified Rankin's Score (mRS) at 3-months post-AIS, length-of-stay (LoS), total hospitalization cost, symptomatic intracerebral hemorrhage (SICH) and inpatient mortality.

**Results:**

1,146 patients (62.0% male) with mean age of 68.6 years were included. Overall, 47.6% of patients achieved a mRS of 0–2 at 3 months after AIS. Patients with DTN time of ≤ 30 min demonstrated higher odds of achieving mRS 0–2 at 3 months (OR 2.35, 95% CI 1.26–4.39) compared to DTN time of ≥60 min. They also experienced 4-day shorter length of stay (LoS) until rehabilitation (*p* = 0.005) and 22.7% reduction in total hospitalization costs (*p* = 0.004).

**Conclusions:**

This study suggests that DTN time of ≤ 30 min is associated with improved functional outcomes and significant cost benefits, supporting consideration of this more aggressive target for acute stroke units. Further research is needed to assess feasibility and broader impact of implementing a 30-min DTN goal in routine clinical practice.

## Introduction

Acute ischaemic stroke (AIS) is the second leading cause of death worldwide (Katan and Luft, 2018), with over 25% of the global adult population expected to experience a stroke in their lifetime (Feigin et al., 2022). Patients who present with a disabling AIS may be eligible for either intravenous thrombolysis (IVT) and/or endovascular thrombectomy (EVT) (Baron et al., 1995). Early administration of IVT has demonstrated efficacy in reducing stroke-related mortality and morbidity (Hacke et al., 2008).

The door-to-needle (DTN) time is defined as the interval between arriving at the hospital and delivering IVT. The current American Heart Association guidelines advocate for stroke centers to attain DTN times of < 60 min for at least 75% of patients due to mortality and morbidity benefits seen at 90 days post-stroke (Powers et al., 2018; Fonarow et al., 2011). Recent studies have highlighted benefits of further reducing DTN time to < 30 min, demonstrating a reduction in both mortality and readmission rates, post-treatment National Institute of Health Stroke Scale (NIHSS) and modified Rankin Scale (mRS) (Man et al., 2020, 2023; Rajan et al., 2021).

In recognition of the importance of rapid thrombolytic intervention, *Target: Stroke Phase III* was introduced by the American Heart Association in 2019 with updated national goals for DTN times. The primary goal is to achieve DTN times within 60 min in at least 85% of acute ischemic stroke patients treated with IV thrombolytics. The secondary goals are to achieve DTN within 45 min in 75% of patients, and within 30 min in 50% of patients. These increasingly stringent benchmarks reflect a national commitment to expedite acute stroke care and improve patient outcomes (Powers et al., 2018).

These improvements in treatment timelines are deeply influenced by efficiency of the entire “stroke chain of survival,” which spans prehospital triage, emergency transport, in-hospital imaging, and prompt therapeutic intervention. In-hospital delays frequently arise from disruptions along this chain, and optimizing each step has been shown to meaningfully shorten DTN times. For instance, models involving direct admission to imaging rooms such as CT or MRI—bypassing emergency department triage—have been associated with expedited treatment and better outcomes (Kamal et al., 2014; Meretoja et al., 2012). Thus, the refinement of stroke care protocols demands a systems-level approach rather than focusing solely on the moment of IVT administration (Puolakka et al., 2016).

Given the heterogeneity of existing results and the predominance of Caucasian cohorts in the literature, a significant limitation is the lack of validation in Asian populations. Reassessing the impact of DTN time < 30 min on AIS outcomes within an Asian cohort could provide valuable insights to inform region-specific clinical practices and stroke care protocols. Additionally, no current studies specifically explore the potential influence of hospitalization costs associated with shorter DTN times. These gaps highlight the need for further research to clarify the clinical benefits, economic and resource allocation implications on hyperacute stroke protocols and healthcare staff within Asian settings.

## Methods

### Study design and data collection

We conducted a single-center observational cohort study on consecutive patients with AIS who underwent IVT therapy (alteplase at 0.9 mg/kg) from January 2017 to December 2023 in a comprehensive stroke center in Singapore. The inclusion criteria were (Katan and Luft, 2018) patients who were treated for AIS with IVT (Feigin et al., 2022), patients presenting within 4.5 h from onset of symptoms (Baron et al., 1995), no contraindications to thrombolysis as outlined by American Heart Association (AHA) guidelines (Adams et al., 1996). The exclusion criteria were patients who had an in-hospital AIS and patients with hemorrhagic stroke at presentation.

Clinical data were collected prospectively in an acute stroke registry and included patient demographics and comorbidities, biochemical and neuroimaging test results, treatment details, quality of care metrics and costs of hospitalization. Demographic data included age, gender and ethnicity. Comorbidities were assessed using the Charlson Comorbidity Index (CCI) (Charlson et al., 1987). Imaging markers, such as the presence of large vessel occlusion, were collected alongside treatment details, including IVT alone and IVT with EVT. Stroke-related indicators, including baseline mRS and at 3 months post-stroke, National Institutes of Health Stroke Scale (NIHSS) on admission and discharge, TOAST classification of stroke types, in-hospital mortality, and presence of symptomatic intracranial hemorrhage (SICH) as defined by the ECASS-2 criteria were also collected (Hacke et al., 1999).

DTN time was defined as the time from arrival of the patient in the emergency department to the time of initiation of IVT. Based on data collected, patients were stratified on their DTN time into 4 groups: (a) < 30 min; (b) 31–45 min; (c) 46–60 min; (d) >60 min.

### Outcome measures

The primary outcome was functional independence, defined as a mRS of 0–2 at 3 months after AIS.

Secondary outcomes included in-hospital mortality, SICH, quality of care metrics including length of stay (LoS) till rehabilitation or discharge, total hospitalization costs and categorical cost per hospitalization. The total and categorical cost to the hospital per patient per hospitalization was also collected. Specific components of categorical costs were elaborated in the Appendix. Cost data assessment was conducted using a value-driven outcome initiative previously published by Tan et al. (2023) which involved standardizing individual cost values to the mean total hospitalization costs of the entire cohort. The mean total hospitalization costs were calculated by summation of the hospitalization costs of the entire cohort, divided by the number of included patients and given an index value of 100.

### Statistical analysis

Frequencies and percentages were used to summarize categorical variables and the Pearson's χ^2^ test for independence was used to compare variables between patient groups of DTN times < 60 min against the reference group of DTN time >60 min. Continuous variables were presented as means with standard deviations (SD) and a *t*-test was utilized to compare groups of patients stratified based on DTN times to the reference group of DTN time >60 min.

Logistic regression models were used to estimate odds ratios (ORs) for the association between DTN times and binary clinical outcomes, including modified Rankin Scale (mRS) scores at 3 months post-stroke and in-hospital mortality. To ensure the patient groups were comparable, we have made further refinement of the study cohort for certain outcomes. Specifically, patients with a baseline mRS >2 were excluded from the mRS analysis, while cases with in-hospital mortality were excluded from models assessing length of stay (LoS) until rehabilitation.

Multivariate linear regressions were performed to evaluate the impact of DTN on hospitalization costs and LoS. To address skewed cost distributions, logarithmic transformation was applied, enabling normalization and proportional interpretation of the results. We have adjusted patients' demographics and comorbidities to control for the confounding effects and enhance the validity and generalizability of our findings.

The analyses were adjusted for potential confounders determined a priori, including demographic factors (age, gender, and race) and clinical variables such as the CCI, smoking status, NIHSS score at admission, treatment with EVT, and the presence of large vessel occlusion. Logistic regression results were expressed as ORs with 95% confidence intervals. All statistical analyses were performed using STATA Version 15.

## Results

### Study demographics

**Figure 1:**
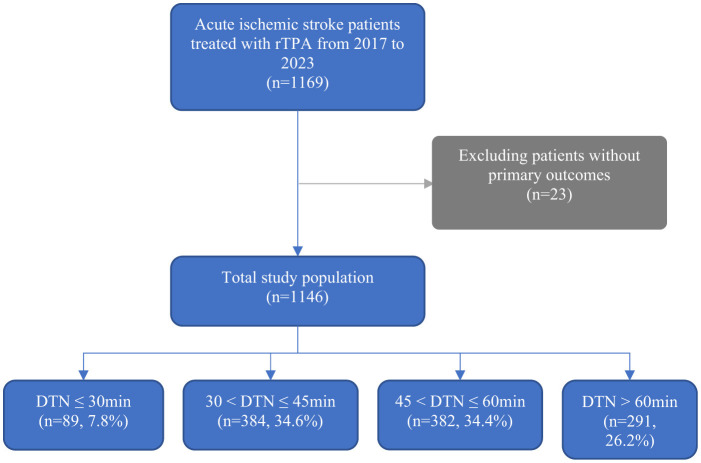
Patients included in the current study from January 2017–December 2023. **Abbreviations** DTN, Door-to-needle time.

A total of 1,169 patients with AIS who received intravenous thrombolysis IVT between January 2017 and December 2023 were initially screened. After excluding 23 patients due to missing primary outcome data, 1,146 patients were included in the final analysis (Figure 1). The cohort comprised 62% male patients, with a mean age of 68.6 years (SD 13.8) (Table 1). Among them, 89 patients (7.8%) achieved a DTN time of < 30 min, 384 patients (34.6%) had a DTN time between 31 and 45 min, 382 patients (34.5%) had a DTN time between 46 and 60 min, and 291 patients (26.2%) had a DTN time >60 min.

**Table 1:** Summary of demographics, co-morbidities, door-to-needle time, stroke-related indicators.

**Variables**	**Overall**	** ≤ 30^a^**	**31–45^a^**	**46–60^a^**	**>60^a^**	***p*-value**
No. of patients, *n* (%)^b^	1146	89 (7.77)	384 (34.6)	382 (34.5)	291 (26.2)	
Age (Mean ± SD) ^b^	68.6 ± 13.8	66.3 ± 13.5	66.7 ± 13.0	69.9 ± 14.4	70.0 ± 13.7	**0.002**
**Gender**, ***n*** **(%)**^b^						
Male	710 (62.0)	66 (74.2)	258 (67.5)	228 (59.4)	158 (54.3)	**< 0.001**
**Ethnicity**, ***n*** **(%)**^b^						
Chinese	761 (66.4)	56 (62.9)	241 (63.1)	271 (70.6)	193 (66.3)	**0.041**
Malay	163 (14.2)	11 (12.4)	39 (10.2)	33 (8.59)	31 (10.7)	
Indian	114 (9.95)	9 (10.1)	61 (16.0)	44 (11.5)	49 (16.8)	
Others^c^	108 (9.42)	13 (14.6)	41 (10.7)	36 (9.38)	18 (6.19)	
**Comorbidities/Risk factors**, ***n*** **(%)**^b^						
Diabetes mellitus	397 (34.6)	35 (39.3)	137 (35.7)	122 (31.9)	103 (35.4)	0.323
Hypertension	863 (75.3)	68 (76.4)	293 (76.3)	277 (72.5)	225 (77.3)	0.127
Hyperlipidaemia	705 (61.5)	60 (67.4)	229 (59.6)	235 (61.5)	181 (62.2)	0.373
Ischemic Heart Disease	208 (18.2)	17 (19.1)	57 (14.8)	70 (18.3)	64 (22.0)	**0.018**
Atrial fibrillation	364 (31.8)	22 (24.7)	103 (26.8)	145 (38.0)	94 (32.3)	0.132
Previous history of TIA/ischemic stroke	155 (13.5)	13 (14.6)	42 (10.9)	49 (12.8)	51 (17.5)	**0.015**
Smoker	181 (15.8)	14 (15.7)	79 (20.6)	53 (13.9)	35 (12.0)	**0.003**
Urinary tract infection	172 (15.0)	8 (8.99)	51 (13.3)	57 (14.9)	56 (19.2)	**0.024**
Pneumonia	170 (14.8)	10 (11.2)	43 (11.2)	59 (15.5)	58 (19.9)	**0.002**
**Stroke-related indicators** ^b^						
mRS on admission ≤ 2, *n* (%)	982 (85.7)	83 (93.3)	345 (90.3)	324 (84.4)	230 (79.0)	**< 0.001**
NIHSS on admission, mean ± SD	12.48 ± 7.84	12.96 ± 8.67	12.00 ± 7.63	12.80 ± 7.66	12.53 ± 8.11	0.385
NIHSS on discharge ≤ 5, *n* (%)	654 (57.0)	55 (61.8)	234 (61.3)	209 (54.4)	156 (53.6)	**0.047**
Large and medium vessel occlusion, *n* (%) ^d^	572 (49.9)	40 (44.9)	190 (49.7)	199 (51.8)	143 (49.1)	0.489
EVT	42 (3.66)	2 (2.25)	13 (3.40)	18 (4.69)	9 (3.09)	0.296
Onset to ED, mean ± SD	105.4 ± 62.1	145.2 ± 67.4	105.7 ± 61.1	101.2 ± 54	98.2 ± 67.1	**< 0.001**
**TOAST classification** ^b^						
1—Large-artery atherosclerosis (LAA)	246 (21.5)	12 (13.5)	94 (24.5)	74 (19.4)	66 (22.7)	**0.013**
2—Cardioembolic	455 (39.7)	34 (38.2)	130 (33.9)	168 (44.0)	123 (42.3)	
3—Small vessel disease	194 (16.9)	20 (22.5)	83 (21.6)	54 (14.1)	37 (12.7)	
4—Stroke of other determined cause	17 (1.48)	1 (1.12)	5 (1.30)	3 (0.79)	8 (2.75)	
5—Stroke of undetermined cause	234 (20.4)	22 (24.7)	70 (18.2)	85 (22.3)	57 (19.6)	

DTN, Door-to-needle time; CCI, Charlson Comorbidity Index; SD, Standard Deviation; mRS, Modified Rankin scale; NIHSS, National Institute of Health Stroke Scale.

^a^Data is expressed as No. (%) unless otherwise stated.

^b^t-tests were adopted to determine the statistical difference of continuous variables between any DTN group versus the baseline group (DTN over 60). Pearson Chi-square tests were adopted to determine the statistical difference of categorical variables.

^c^Others include Eurasians, Sikh, and other race categories.

^d^Medium vessel occlusions were defined as occlusions in non-dominant M2, M3, A1, A2, P1 or P2 (Saver et al., 2020).

All bolded texts are significant values.

### Primary outcome

**Figure 2:**
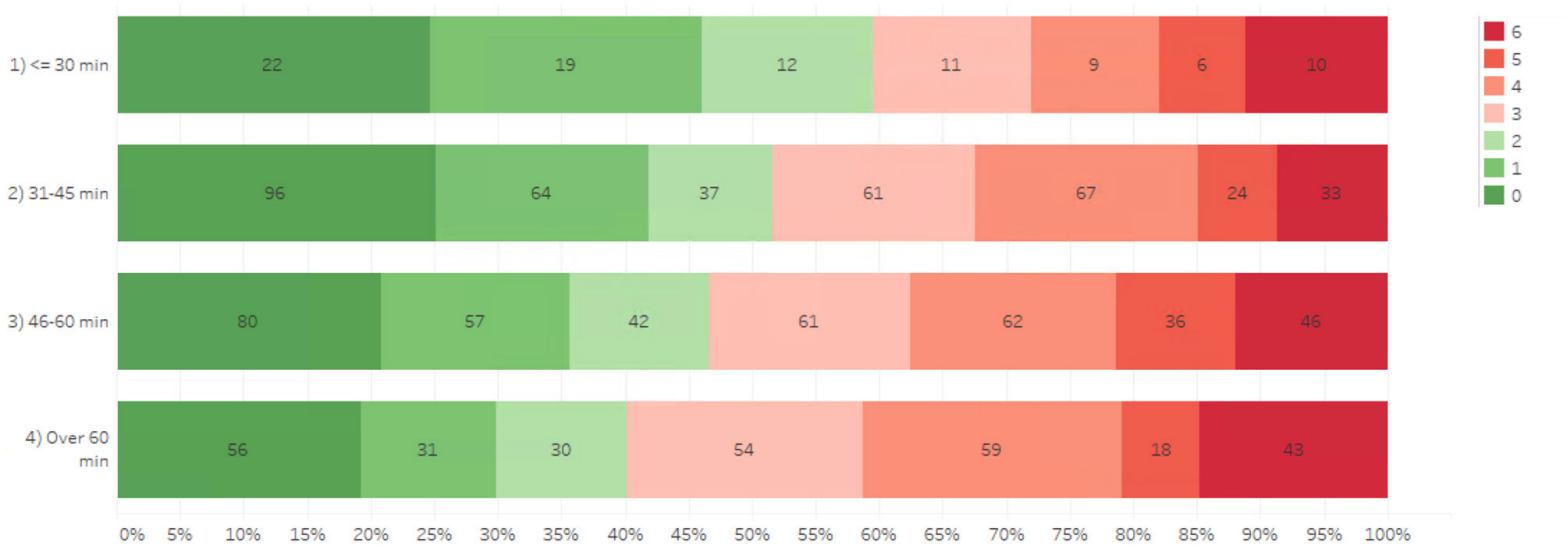
Association of door-to-needle time and mRS 3-months post-stroke.

The association between DTN times and mRS at 3 months post-stroke are shown in Figure 2. Model-free evidence suggests that the proportion of patients achieving favorable functional outcomes after AIS decreases progressively with longer DTN times.

The association of DTN with primary and secondary outcomes are shown in Table 2. The model estimation results revealed that DTN time ≤ 30 min was significantly associated with better outcomes. Specifically, patients with DTN time ≤ 30 min demonstrated significantly higher odds of functional independence at 3-months compared to DTN >60 min (OR 2.35, Confidence Interval [CI] 1.26–4.39, *p* = 0.007) in Table 2.

**Table 2:** Association of DTN time with primary and secondary outcomes.

**Variables**		**mRS ≤ 2 at 3 months^a^**	**In-hospital Mortality^a^**	**LoS Till Rehab^b^**	**SICH^a^**	**Total hospitalization costs^b^**
**Adjusted Odds Ratio/Estimators** ^c^						
DTN ≤ 30	OR (95% CI)	**2.35 (1.26–4.39)**	0.833 (0.329–2.11)	**−4.15 (−7.05–1.26)**	0.216 (0.026–1.81)	**−0.227 (−0.382–0.073)**
	*p*	**0.007**	0.669	**0.005**	0.158	**0.004**
DTN 31–45	OR (95% CI)	1.20 (0.809–1.79)	0.651 (0.346–1.23)	−1.51 (−3.34–0.326)	0.974 (0.409–2.32)	−0.022 (−0.120–0.076)
	*p*	0.361	0.183	0.107	0.952	0.663
DTN 46–60	OR (95% CI)	1.21 (0.809–1.81)	0.868 (0.490–1.54)	−1.84 (−3.67–0.001)	0.889 (0.377–2.10)	−0.053 (−0.151–0.045)
	*p*	0.355	0.626	0.0501	0.789	0.287
DTN > 60	Reference group					

mRS, Modified Rankin scale; LoS, Length of stay; SICH, symptomatic intracranial hemorrhage; OR, Odds ratio; CI, 95% Confidence Interval; *p, p*-value.

^a^Logistic regressions are performed for binary outcomes, including mRS, mortality and SICH.

CI in e-form (for logistic regressions) are in parentheses.

^b^Linear regressions are performed for continuous outcomes, including LoS and cost savings.

CI (for linear regressions) are in parentheses.

^c^Model adjusted for variables including patient age on admission, race, gender, smoker, Charlson comorbidity index (CCI), National Institute Health Stroke Scale (NIHSS) on admission, large vessel occlusion, and endovascular thrombectomy. In the models for mRS-3m (0–2), cases with mRS on admission over 2 were excluded. In the models for LoS till rehabilitation, cases with in-hospital mortality were excluded from the analysis.

All bolded texts are significant values.

### Secondary outcomes

DTN ≤ 30 min was also significantly associated with a 4-day reduction of LoS till rehabilitation (*p* = 0.004) and a reduction of 22.7% in total hospitalization costs (*p* = 0.004), compared to DTN times over 60 min as demonstrated in Table 2. SICH and in-hospital mortality were not significantly associated with DTN time on logistic regression.

## Discussion

In our study, we found that 73.8% of our cohort met AHA recommendation of receiving IVT within 60 min. Notably, 7.8% of patients achieved a DTN time within 30 min. While our current institutional target is a DTN of 60 min, the potential benefits observed with shorter DTN times suggest that revising these targets may be worth evaluating.

Specifically, we found that DTN time ≤ 30 min was significantly associated with functional independence at 90 days post-stroke, as well as a 4-day reduction in length of stay (*p* = 0.004). These findings support the principle of “time is brain,” where earlier IVT administration results in faster restoration of perfusion and the preservation of at-risk brain tissue (Saver, 2006). Importantly, our study did not observe a ceiling effect, meaning that even the shortest DTN times ( ≤ 30 min) continued to yield significant clinical and functional benefits, with no apparent diminishing returns in terms of improved outcomes. Our results are consistent with existing literature, which shows that shorter DTN times, especially those under 30 min, are associated with improved mRS scores and reduced LoS (Man et al., 2023; Rajan et al., 2021).

Our findings suggest that a more stringent target of DTN ≤ 30 min may result in even better outcomes compared to the current AHA recommendation (Powers et al., 2018). However, key limitations remain, particularly in developing effective strategies to further reduce DTN times. To achieve this, it is essential to enhance the current triage systems and improve response times of stroke units.

In our center, several strategies are employed to reduce DTN times (Katan and Luft, 2018): prenotification by paramedics (Feigin et al., 2022), direct transfer from ambulance to CT scanner (Baron et al., 1995), rapid en-route neurological assessment by both an emergency physician and neurologist (Hacke et al., 2008), the presence of a specialized stroke nurse accompanying the patient to expedite various steps in the treatment pathway, including facilitating endovascular treatment in eligible cases and (Powers et al., 2018) use of Rapid.AI to aid in the diagnosis (Tan et al., 2018).

Looking ahead, newer approaches could be trialed to meet the proposed target of DTN ≤ 30 min. These may include simple interventions such as administering IVT at the CT scanner, or pre-diluting IVT medications (Kamal et al., 2017; Siarkowski et al., 2020). Additionally, more complex strategies, such as AI-automated solutions for ischemic stroke detection could reduce decision-making and treatment time (Temmen et al., 2023). Another promising model that has gained traction is the “drip-and-ship” protocol. In this approach, patients eligible for both IVT and EVT receive thrombolytic therapy at the presenting emergency department while awaiting transfer to a tertiary center capable of EVT, thereby reducing delays to treatment (de la Ossa et al., 2022).

Rather than adopting blanket prescriptions that may not be suitable for all institutions, it is crucial for individual hospitals to conduct regular audits. These audits can identify potential breaks in the care chain that contribute to unnecessary prolongation of DTN times, thus enabling tailored interventions that can streamline treatment and improve patient outcomes. It is also critical to acknowledge that prioritizing stroke cases often requires significant resource allocation, which can strain other areas of care delivery. This reallocation may incur indirect costs not captured in this study, potentially affecting the overall efficiency of the healthcare system. These factors must be carefully considered when implementing stricter DTN targets (Meretoja et al., 2013).

Our study found that a DTN time >60 min was not associated with an increased risk of symptomatic intracerebral hemorrhage (SICH) compared to DTN time ≤ 30 min. During strokes, hypoxia in the ischemic core leads to a cellular cascade that damages the BBB (Flick, 2021). Prolonged ischemia exacerbates this damage, making the BBB more permeable and increasing the risk of hemorrhage when tPA is administered (Flick, 2021). However, if tPA is given within the 4.5-h window, the likelihood of extensive BBB disruption is reduced, as there is still sufficient viable penumbra that can be salvaged without causing excessive reperfusion injury (Yang and Liu, 2021).

In our study, patients with DTN times >60 min likely benefited from this effect because the extent of ischemic damage was still within a manageable threshold. However, it is possible that the current DTN stratification was not sensitive enough to detect subtle differences in risk—finer groupings, such as DTN >90 min, might reveal greater associations between delayed treatment and SICH (Flick, 2021). Moreover, it is important to consider that expediency in decision-making can lead to an increased likelihood of missing contraindications, such as subtle hemorrhages, which could have critical consequences. A nuanced discussion of these trade-offs is necessary to fully appreciate the complexities involved in optimizing DTN for acute stroke management even though it did not demonstrate an increase of risk in this study.

To our knowledge, this study is the first to link DTN ≤ 30 min to lower cost of hospitalization for the care providers (22.7%, *p* = 0.004). Previous studies have not shown significant cost savings or have not investigated this aspect (Man et al., 2023; Rajan et al., 2021; Kruyt et al., 2013). These savings were heavily driven by a shorter LoS as outlined in Table 3 with a significantly lower average room charge, daily treatment fees and investigation fees. It is also worth noting that rates of nosocomial infections like urinary tract infections or pneumonia were significantly lower in the DTN ≤ 30 min group (Table 1). The association between an increased length of stay and a greater risk of nosocomial infections is well-known and likely contributes to a vicious circle, thereby increasing healthcare cost (Zimlichman et al., 2013). Improved functional outcomes also result in earlier mobilization and a reduction in the amount of time the patient stays bedbound, which reduces the risk of nosocomial infections. These findings provide a strong impetus for stroke units to implement more ambitious DTN targets that can contribute to a shorter LoS.

**Table 3:** Summary of primary and secondary outcomes.

**Door-to-needle time, min**						
**Variables**	**Overall**	≤ **30**^a^	**31–45** ^a^	**46–60** ^a^	>**60**^a^	***p*** **-value** ^b^
**Outcomes**						
mRS ≤ 2 at 3 months post stroke	546 (47.6)	53 (59.6)	197 (51.6)	179 (46.6)	117 (40.2)	**0.001**
LoS mean ± SD, days	12.3 ± 14.7	9.28 ± 9.55	11.7 ± 14.5	12.4 ± 12.5	13.8 ± 18.5	**0.027**
In-hospital mortality	93 (8.12)	8 (8.99)	22 (5.76)	33 (8.59)	30 (10.3)	**0.029**
SICH	38 (3.32)	1 (1.12)	13 (3.40)	12 (3.13)	12 (4.12)	0.174
**Cost, mean (SD), % of average total hospitalization costs**						
Total hospitalization costs^c^	100 (98.8)	78.7 (58.2)	101 (110.6)	99.3 (83.1)	106.3 (110)	**0.024**

SD, Standard Deviation; mRS, Modified Rankin scale; LoS, Length of stay; SICH, symptomatic intracranial hemorrhage.

^a^Data is expressed as No. (%) unless otherwise stated.

^b^t-tests were adopted to determine the statistical difference of continuous variables between any DTN group vs. the baseline group (DTN over 60). Pearson Chi-square tests were adopted to determine the statistical difference of categorical variables.

^c^All costs were calculated against a pre-determined index which is outlined in the methodology section.

All bolded texts are significant values.

Our study contrasts with previous literature that primarily examined healthcare systems characterized by a mix of private insurance and public-funded health coverage. In those settings, achieving door-to-needle (DTN) times of ≤ 30 min did not consistently demonstrate cost savings (Rajan et al., 2021). In Singapore's healthcare model, however, the co-payment system—underpinned by government subsidies and the “3M” framework (Medisave, MediShield Life, and Medifund)—places a stronger emphasis on cost-efficiency and affordability. This model ensures that patients contribute to their care costs, but significant government subsidies and means testing help keep these contributions manageable, particularly for lower-income groups.

Reducing DTN times can lead to substantial clinical and financial benefits. Faster intervention for conditions like acute ischemic stroke minimizes complications, shortens hospital stays, and reduces the likelihood of long-term disability, which directly correlates with lower healthcare costs. This not only benefits the patients but also aligns with the broader goals of Singapore's healthcare policy, which seeks to maintain high standards of care while managing costs effectively. Our study provides evidence that, within this co-payment model, there are clear incentives for healthcare providers to adopt practices that lead to better outcomes without escalating costs, reinforcing the critical need for prompt and efficient acute care interventions.

A key strength of this study is the inclusion of a large number of patients from a predominantly South-East Asian population. To our knowledge, this is the first study that demonstrates an improvement in outcomes from a shortened DTN time in such a population. It is also notable for showing an association between shorter DTN times and shorter LoS. Moreover, the effects of DTN was independent of time of onset to ED. In fact, the onset to ED time was longer for patients with DTN time < 30 min likely due to urgency of administering rTPA to patients who are close to 4.5 h threshold.

An important limitation is that here were some dissimilarities across—most notably, the patients who had DTN ≤ 30 min also tended to have significantly higher rates of functional independence. Moreover, another limitation is the relatively low proportion of patients who received EVT, as the cohort included patients with low ASPECTs or low NIHSS which were deemed unsuitable for EVT. This has been adjusted for. We also acknowledge the limitation of focusing primarily on DTN rather than total ischemic time, which includes prehospital delays; however, DTN remains a modifiable in-hospital metric that healthcare systems can directly influence to improve outcomes.

Future studies may include longer-term follow-up to determine the impact of shorter DTN times on other outcomes beyond functional independence, particularly in less-commonly explored areas such as cognition or gait impairments from vascular dementia and Parkinsonism (Wang et al., 2023, 2025a,b).

Our study provides further impetus for acute stroke units to continually self-audit and seek best practices in identifying bottlenecks and lowering DTN time. While it is unlikely that a cookie-cutter approach exists to overcoming local and structural factors at each individual center, available literature suggests that many centers may address low-hanging fruit such as streamlining triage or automated in-hospital alert systems (Kruyt et al., 2013).

## Conclusion

This current study highlights the significant benefits of further reducing the DTN target for ischemic stroke patients treated with intravenous thrombolysis. Our findings suggest a DTN time ≤ 30 min is associated with better functional outcomes (e.g., better mRS scores) and a shortened length of stay which translates into considerable cost savings for the healthcare providers.

These results provide an impetus to healthcare systems and administrators to continue to pursue both practical and strategic measures in striving to lower DTN times. The practical implementation of more ambitious targets remains an important area of further study.

## Data Availability

The raw data supporting the conclusions of this article will be made available by the authors, without undue reservation.
