# Supplementary material for: Improved functional outcomes and cost benefits of door-to-needle time under 30 min in acute ischemic stroke: an observational study
Source: Front Stroke. 2025 Jun 3;4:1583875. doi: 10.3389/fstro.2025.1583875 (PMC12802697; doi:10.3389/fstro.2025.1583875)
Supplement: Supplementary file 1 [file Data_Sheet_1.docx]

# Appendix

*Table: Breakdown of Categorical Costs*

| **Categories** | **Components** |
| --- | --- |
| Room charges | Clinic Procedure Facility Charge (ICU, HD, Class A, B and C) |
| Consumables | Consumables |
| Investigations | X-ray  Laboratory  Magnetic Resonance Imaging (MRI)  Specialised |
| Medications | Non Standard  Standard |
| Daily treatment fees | Ward Bed Operational Charges (ICU, HD, Class A, B and C) |
| Therapy services | Speech Therapy  Occupational Therapy  Physiotherapy  Other Therapy |
| Treatment services | Ward/ Clinic / Other Procedures  Assessment & Counselling  Other Treatment Services  Renal Dialysis  A&E fee Transfer to IP |
| Surgery services | Surgical Procedure  Implant |

*Table: Breakdown of cost of total hospitalisation*

| **Cost, mean (SD), % of average total hospitalisation costs** | | | | | | | |
| --- | --- | --- | --- | --- | --- | --- | --- |
| Total hospitalisation costs | 100 (98.8) | 78.7 (58.2) | 101 (110.6) | 99.3 (83.1) | 106.3 (110) |  | **0.024** |
| Room charges | 34.4 (42.1) | 24.9 (22.7) | 33.3 (44.1) | 34.3 (35.2) | 38.8 (51.1) |  | **0.014** |
| Consumables | 4.17 (6.69) | 4.03 (6.50) | 4.60 (7.04) | 4.28 (6.66) | 3.52 (6.28) |  | **0.038** |
| Investigations | 12.3  (8.19) | 9.50 (4.79) | 12.2 (8.71) | 12.5 (8.17) | 13.0 (8.18) |  | **<0.001** |
| Medications | 8.7 (3.57) | 8.07 (3.03) | 9.01 (3.63) | 8.59 (3.39) | 8.63 (3.83) |  | 0.191 |
| Daily treatment fees | 17.3 (19.5) | 12.6 (11.7) | 17.1 (22.7) | 17.2 (17.2) | 19.0 (19.7) |  | **0.004** |
| Therapy services | 4.67 (7.66) | 3.93 (6.42) | 4.53 (6.31) | 4.53 (6.77) | 5.27 (10.3) |  | 0.249 |
| Treatment services | 6.45 (13.6) | 5.46 (12.6) | 6.57 (17.8) | 5.98 (8.54) | 7.24 (13.0) |  | 0.132 |
| Surgery services | 10.9 (18.3) | 9.42 (15.9) | 12.6 (19.2) | 11.0 (18.3) | 9.18 (17.4) |  | **0.018** |

*Table: Unadjusted Odds Ratio/Estimates of outcomes*

|  | **Unadjusted Odds Ratio/Estimators** | | | | | |
| --- | --- | --- | --- | --- | --- | --- |
|  |  | mRS ≤2 at 3 months^a^ | In-hospital Mortality^a^ | LoS Till Rehab^b^ | SICH^a^ | Total hospitalisation costs^b^ |
| DTN <= 30 | OR | **1.74** | 0.859 | **-4.02** | 0.264 | **-0.217** |
|  | CI | **1.04 - 2.91** | 0.379 - 1.95 | **-6.93 - -1.11** | 0.034 - 2.06 | **-0.393- -0.042** |
|  | p | **0.036** | 0.717 | **0.007** | 0.204 | **0.015** |
| DTN 31-45 | OR | 1.28 | **0.530** | -1.54 | 0.819 | -0.035 |
|  | CI | 0.914 - 1.79 | **0.300 - 0.943** | -3.41 - 0.325 | 0.368 - 1.83 | -0.148 - 0.078 |
|  | p | 0.152 | **0.031** | 0.105 | 0.625 | 0.543 |
| DTN 46-60 | OR | 1.15 | 0.818 | -1.38 | 0.750 | -0.011 |
|  | CI | 0.823 - 1.62 | 0.486 - 1.38 | -3.24 - 0.484 | 0.332 - 1.69 | -0.123 -0.102 |
|  | p | 0.406 | 0.449 | 0.147 | 0.489 | 0.851 |
| DTN > 60 | *Reference group* | | | | | |
|  | | | | | | |

*Table: Association of DTN and cost*

| \|  \| **Unadjusted Estimators** \| \| \| \| \| \| \| \| \| \| --- \| --- \| --- \| --- \| --- \| --- \| --- \| --- \| --- \| --- \| \| VARIABLES \| Total hospitalisation costs \| RoomCharge \| Consumables \| Investigations \| Medications \| DailyTreatmentFee \| TherapyServices \| TreatmentServices \| SurgeryService \| \| DTN <= 30 \| -0.217* \| -0.397*** \| -0.200 \| -0.350*** \| -0.185* \| -0.383*** \| -0.642*** \| -0.363* \| 0.642 \| \| (-0.393 - -0.042) \| (-0.608 - -0.185) \| (-0.635 - 0.235) \| (-0.492 - -0.207) \| (-0.337 - -0.034) \| (-0.595 - -0.171) \| (-1.070 - -0.214) \| (-0.662 - -0.064) \| (-0.366 - 1.650) \| \| 0.015 \| 0.000 \| 0.366 \| 0.000 \| 0.016 \| 0.000 \| 0.003 \| 0.017 \| 0.211 \| \| DTN 31-45 \| -0.035 \| -0.164* \| 0.243 \| -0.076 \| 0.087 \| -0.133 \| 0.033 \| -0.151 \| 0.695* \| \| (-0.148 - 0.078) \| (-0.300 - -0.028) \| (-0.036 - 0.522) \| (-0.168 - 0.015) \| (-0.010 - 0.184) \| (-0.269 - 0.003) \| (-0.241 - 0.308) \| (-0.343 - 0.041) \| (0.047 - 1.342) \| \| 0.543 \| 0.018 \| 0.088 \| 0.101 \| 0.079 \| 0.055 \| 0.811 \| 0.123 \| 0.035 \| \| DTN 46-60 \| -0.011 \| -0.087 \| 0.288* \| -0.043 \| 0.010 \| -0.085 \| 0.003 \| -0.150 \| 0.426 \| \| (-0.123 - 0.102) \| (-0.222 - 0.049) \| (0.009 - 0.567) \| (-0.135 - 0.048) \| (-0.087 - 0.107) \| (-0.221 - 0.051) \| (-0.271 - 0.278) \| (-0.341 - 0.042) \| (-0.221 - 1.073) \| \| 0.851 \| 0.211 \| 0.043 \| 0.351 \| 0.833 \| 0.221 \| 0.981 \| 0.126 \| 0.197 \| \| DTN > 60 \| *Reference Group* \| \| \| \| \| \| \| \| \| \|  \|  \|  \|  \|  \|  \|  \|  \|  \|  \| \|  \| **Adjusted Estimators** \| \| \| \| \| \| \| \| \| \| VARIABLES \| TotalCost \| RoomCharge \| Consumables \| Investigations \| Medications \| DailyTreatmentFee \| TherapyServices \| TreatmentServices \| SurgeryService \| \| DTN <= 30 \| -0.225*** \| -0.397*** \| -0.164 \| -0.365*** \| -0.208** \| -0.389*** \| -0.634*** \| -0.362** \| 0.629 \| \| (-0.372 - -0.078) \| (-0.587 - -0.207) \| (-0.506 - 0.178) \| (-0.494 - -0.236) \| (-0.358 - -0.058) \| (-0.582 - -0.196) \| (-1.067 - -0.202) \| (-0.615 - -0.110) \| (-0.201 - 1.458) \| \| 0.003 \| 0.000 \| 0.348 \| 0.000 \| 0.007 \| 0.000 \| 0.004 \| 0.005 \| 0.137 \| \| DTN 31-45 \| -0.004 \| -0.111 \| 0.320*** \| -0.063 \| 0.082 \| -0.094 \| 0.032 \| -0.065 \| 0.749** \| \| (-0.098 - 0.091) \| (-0.234 - 0.011) \| (0.100 - 0.540) \| (-0.146 - 0.020) \| (-0.014 - 0.179) \| (-0.218 - 0.030) \| (-0.247 - 0.310) \| (-0.228 - 0.098) \| (0.214 - 1.283) \| \| 0.939 \| 0.075 \| 0.004 \| 0.136 \| 0.095 \| 0.139 \| 0.824 \| 0.434 \| 0.006 \| \| DTN 46-60 \| -0.024 \| -0.089 \| 0.227* \| -0.052 \| 0.014 \| -0.088 \| 0.012 \| -0.152 \| 0.273 \| \| (-0.118 - 0.070) \| (-0.210 - 0.033) \| (0.009 - 0.445) \| (-0.134 - 0.031) \| (-0.082 - 0.109) \| (-0.211 - 0.035) \| (-0.265 - 0.288) \| (-0.314 - 0.009) \| (-0.257 - 0.802) \| \| 0.613 \| 0.152 \| 0.042 \| 0.218 \| 0.779 \| 0.161 \| 0.934 \| 0.064 \| 0.312 \| \| DTN > 60 \| *Reference Group* \| \| \| \| \| \| \| \| \|   Note: CI is included in parentheses. P-value is noted below CI. All cost, including total hospitalisation costs and categorical cost, is logarithm transformed. |
| --- | --- | --- | --- | --- | --- | --- | --- | --- | --- | --- | --- | --- | --- | --- | --- | --- | --- | --- | --- | --- | --- | --- | --- | --- | --- | --- | --- | --- | --- | --- | --- | --- | --- | --- | --- | --- | --- | --- | --- | --- | --- | --- | --- | --- | --- | --- | --- | --- | --- | --- | --- | --- | --- | --- | --- | --- | --- | --- | --- | --- | --- | --- | --- | --- | --- | --- | --- | --- | --- | --- | --- | --- | --- | --- | --- | --- | --- | --- | --- | --- | --- | --- | --- | --- | --- | --- | --- | --- | --- | --- | --- | --- | --- | --- | --- | --- | --- | --- | --- | --- | --- | --- | --- | --- | --- | --- | --- | --- | --- | --- | --- | --- | --- | --- | --- | --- | --- | --- | --- | --- | --- | --- | --- | --- | --- | --- | --- | --- | --- | --- | --- | --- | --- | --- | --- | --- | --- | --- | --- | --- | --- | --- | --- | --- | --- | --- | --- | --- | --- | --- | --- | --- | --- | --- | --- | --- | --- | --- | --- | --- | --- | --- | --- | --- | --- | --- | --- | --- | --- | --- | --- | --- | --- | --- | --- | --- | --- | --- | --- | --- | --- | --- | --- | --- | --- | --- | --- | --- | --- | --- | --- | --- | --- | --- | --- | --- | --- | --- | --- | --- | --- | --- | --- | --- | --- | --- | --- | --- | --- | --- | --- | --- | --- | --- | --- | --- | --- | --- | --- | --- | --- | --- | --- | --- | --- | --- | --- | --- | --- | --- | --- | --- | --- | --- | --- | --- | --- | --- |
